# Geographical distribution of authorship for leading cardiothoracic surgery journals

**DOI:** 10.1111/jocs.17022

**Published:** 2022-10-13

**Authors:** Aashray K. Gupta, Christopher D. Ovenden, Kayla Nathin, Nidhi Aujayeb, Joseph N. Hewitt, Joshua G. Kovoor, Justin C. Y. Chan, Adam Wells

**Affiliations:** ^1^ Discipline of Surgery University of Adelaide Adelaide South Australia Australia; ^2^ Department of Cardiothoracic Surgery New York University Langone Health New York USA

**Keywords:** cardiovascular research, cardiovascular pathology

## Abstract

**Background:**

Evolution of surgical practice is influenced by publications in the leading journals of that field. If the authorship of a publication lacks geographical diversity, this could create bias and limit generalizability of the evidence. Accordingly, we conducted a geographical analysis of the leading Cardiothoracic Surgery journals worldwide.

**Methods:**

Using 2020 Impact Factor, we searched the leading Cardiothoracic Surgery journals over the past decade. Only original articles were included. Data regarding first, second and last authors were extracted from every article. From this, we analysed country of affiliation, highest academic degree obtained and author location by metropolitan or rural setting.

**Results:**

A total of 12,706 original articles were published in the top 5 ranked Cardiothoracic journals between 2011 and 2020. Authors originated from 69 countries, with the majority being from North America and Western Europe. The United States was the most common country of affiliation (42.8%) in all five journals, with New York City the most prominent city. A total of  63.7% of the authorship originated from large metropolitan areas (estimated as population greater than 500,000 residents), and the most common degrees obtained by authors were MD and PhD.

**Conclusion:**

The prominent Cardiothoracic authorship is predominantly located in Western countries, most commonly large metropolitan centers in the United States. This raises questions as to whether the literature adequately reflects populations in other geographical areas such as the continents of South America and Africa and rural settings. Leading journals should consider policies which encourage publication by authors from geographical locations that are underrepresented globally.

## 1 INTRODUCTION

Evolution of surgical practice is influenced by publications in the leading journals of that field. The geographical distribution of publications indicates the influence of regions, countries, cities and even individual institutions on the literature. If the authorship of the literature lacks geographical breadth, this could create bias and limit generalizability of the evidence. A journal's impact factor is an index which measures the average number of citations in a given year for articles published in a journal.^1^ Although there are many metrics to determine the most impactful individual publications, impact factor is widely regarded as an accurate measure to identify the most impactful and influential journals in a field.

In recent years, cardiothoracic surgery has undergone an evolution. Success rates are ever increasing with lower complications despite progressively older and more comorbid patients.^2^, ^3^, ^4^, ^5^, ^6^, ^7^, ^8^ This can be attributed to interventional advances such as minimally invasive, transcatheter and thoracoscopic techniques as well as improved periprocedural care. Given recent advances in the cardiothoracic literature, and changing population demographics worldwide, it is important that the literature published is applicable to all populations.^9^, ^10^, ^11^ To gain an understanding of where the prominent cardiothoracic authorship is located, we conducted a geographical analysis of the leading journals in the field of cardiothoracic surgery.

## 2 METHODS

We used Clarivate Analytics Journal Citation Reports to identify current single year impact factor for journals.^12^ We identified and then searched the top 5 journals ranked by current single year impact factor in the field of cardiothoracic surgery. We analysed all original articles published in these journals for a period of 10 years between 2011 and 2020 inclusive. Data regarding first, second and last authors were extracted from every article. From this, we analysed the country and city of affiliation, highest academic degree obtained and whether the authors were located in a metropolitan or rural setting. We used the UN World Urbanization project Data^13^ to dichotomize cities to a population greater or less than 500,000 in the year 2020.

For further comparison, The Journal of Cardiothoracic Surgery (JCS) and Annals of Thoracic and Cardiovascular Surgery (ATCS) are the top two ranked open access journals by impact factor in the field. Both have the editorial policy of fee waivers for authors from developing countries to encourage publication. We reviewed the geographical distribution of authorship in these journals for the years 2019 and 2020 to assess if fee‐waiver policy addressed this issue.

### 2.1 Data visualization

The number of authors per country are presented graphically. Number of authors per country were first converted to a logarithmic scale in base 10. We then used Microsoft Excel (ver. 16.5, Microsoft Corporation, 2018)^14^ to create heat maps of author distribution by country. These are presented by journal and in total.

The number of authors by city of origin are also presented in heat maps created using Maptive^15^ for three regions, North America, Western Europe and Asia.

## 3 RESULTS

The top 5 ranked journals identified by impact factor were Journal of Heart and Lung Transplantation (JHLT, IF 10.25), Journal of Thoracic and Cardiovascular Surgery (JTCVS, IF 5.21), Annals of Thoracic Surgery (ATS, IF 4.33), European Journal of Cardiothoracic Surgery (JTCVS, IF 4.19) and Annals of Cardiothoracic Surgery (ACS, 4.10).

**Figure 1:**
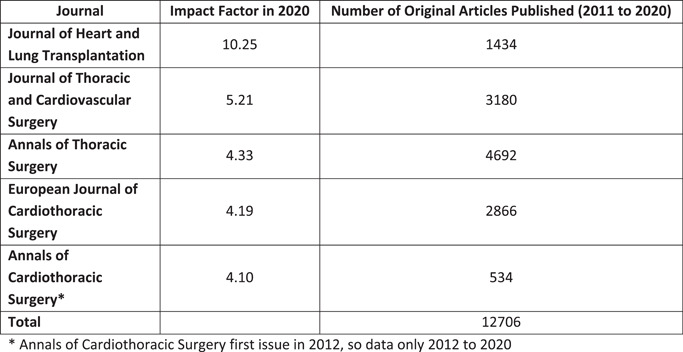
Total Articles by Journal. *Annals of Cardiothoracic Surgery first issue in 2012, so data only 2012 to 2020

**Figure 2:**
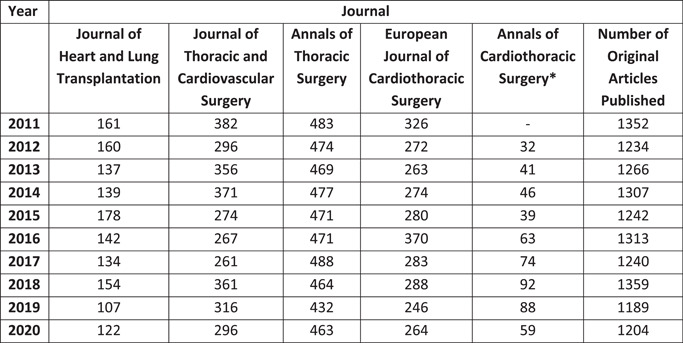
Total articles by year

Our searches retrieved a total of 12,706 articles for analysis. Figure 1 demonstrates the impact factor and total number of publications by each journal across the specified study period. Annals of Thoracic Surgery had the highest number of total publications (*n* = 4692), followed by the Journal of Thoracic and Cardiovascular Surgery (*n* = 3180). Annals of Cardiothoracic Surgery had the lowest volume (*n* = 534), although its first edition was in May 2012, compared with the other journals which predated 2011. Figure 2 demonstrates the number of publications each year by each journal.

**Figure 3:**
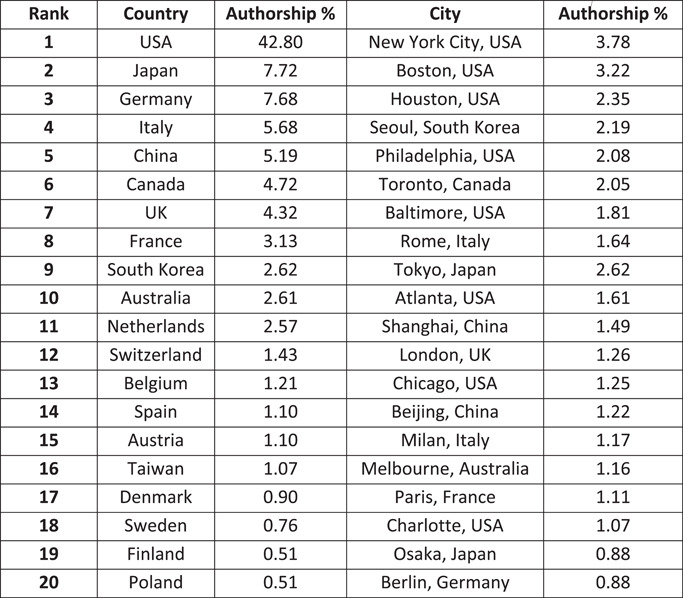
Geographical distribution of authorship

Authors originated from a total of 69 countries. Figure 3 lists the top 20 most common countries of affiliation for authors. The proportion of the total number of publications made up by each country is also given. The same figures are provided showing top 20 cities of author affiliation. The most common cities of authorship were New York (3.8%) United States, Boston (3.2%) United States, Houston (2.4%) United States, Seoul (2.2%) South Korea, and Philadelphia (2.1%) United States. Of the top 20 cities, 8 were located in the United States.

**Figure 4:**
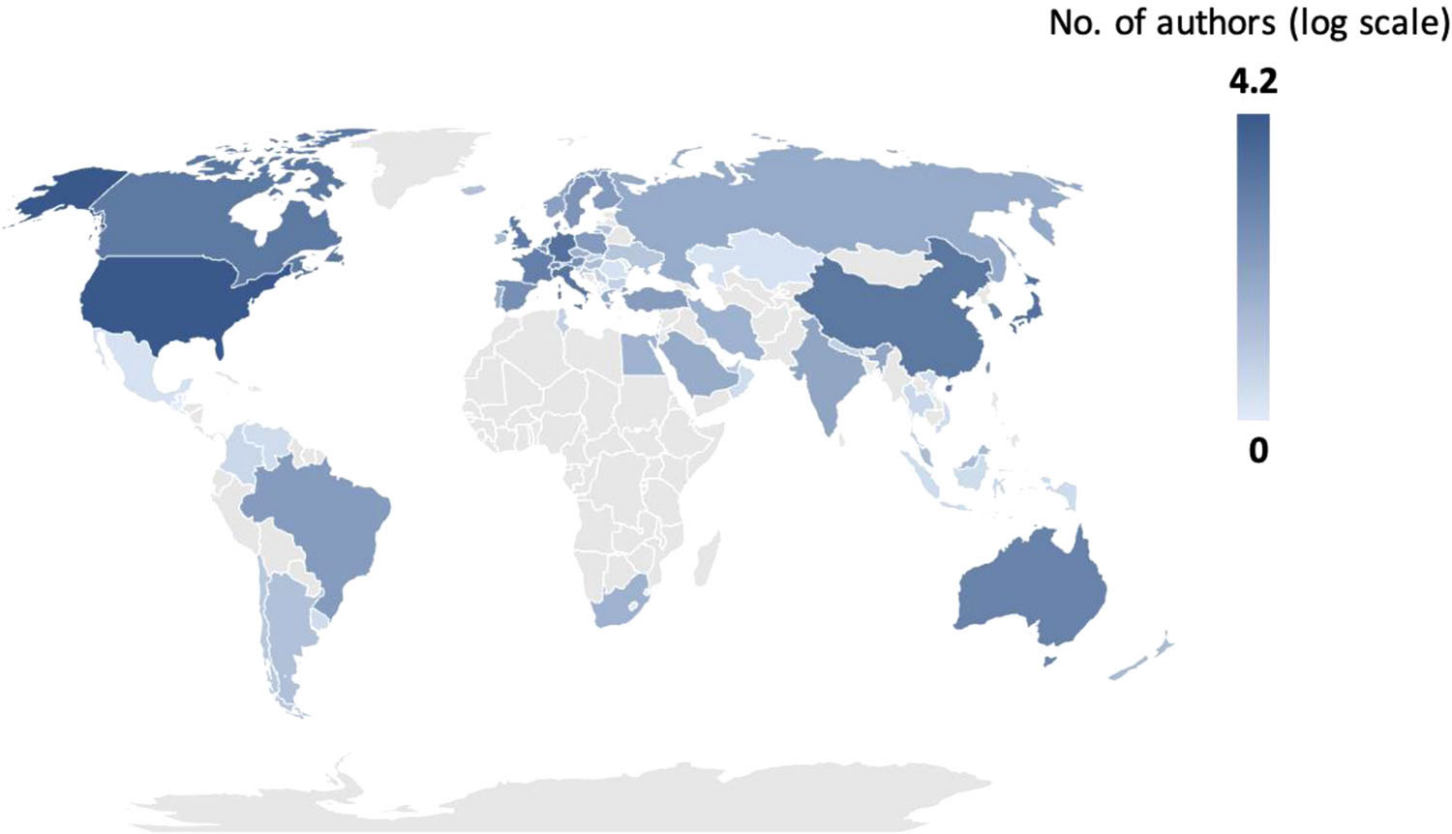
Geographical distribution of authorship for leading Cardiothoracic Surgery Journals

**Figure 5:**
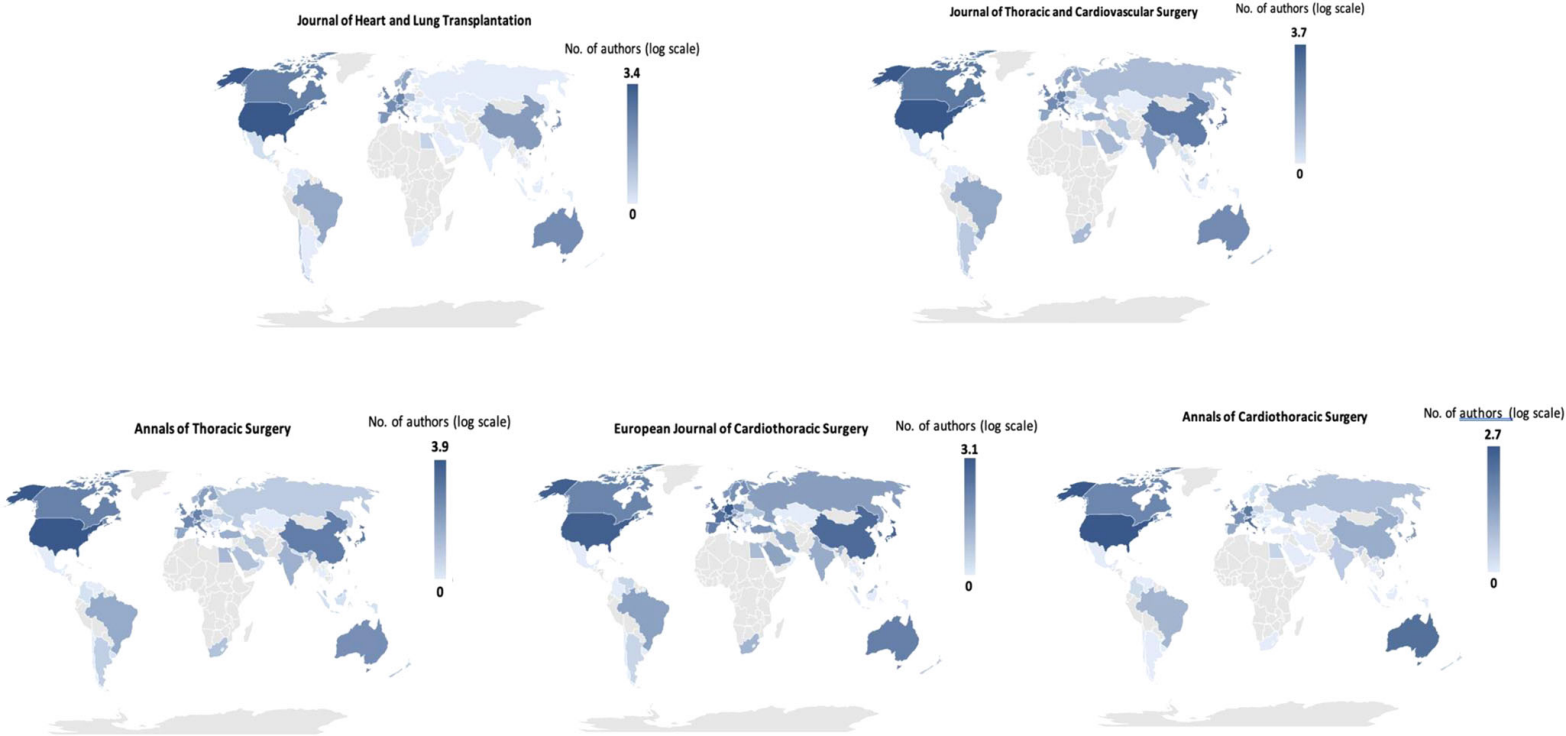
Geographical distribution of authorship for each of top 5 ranked Cardiothoracic Surgery Journals

Figure 4 highlights the concentration of authorship by country using a logarithmic scale. The most common countries of authorship affiliation were the United States (42.8%), Japan (7.7%), Germany (7.7%), Italy (5.7%), and China (5.2%). Figure 5 shows the concentration of authorship affiliation in each of the five journals, visually illustrating the differences.

**Figure 6:**
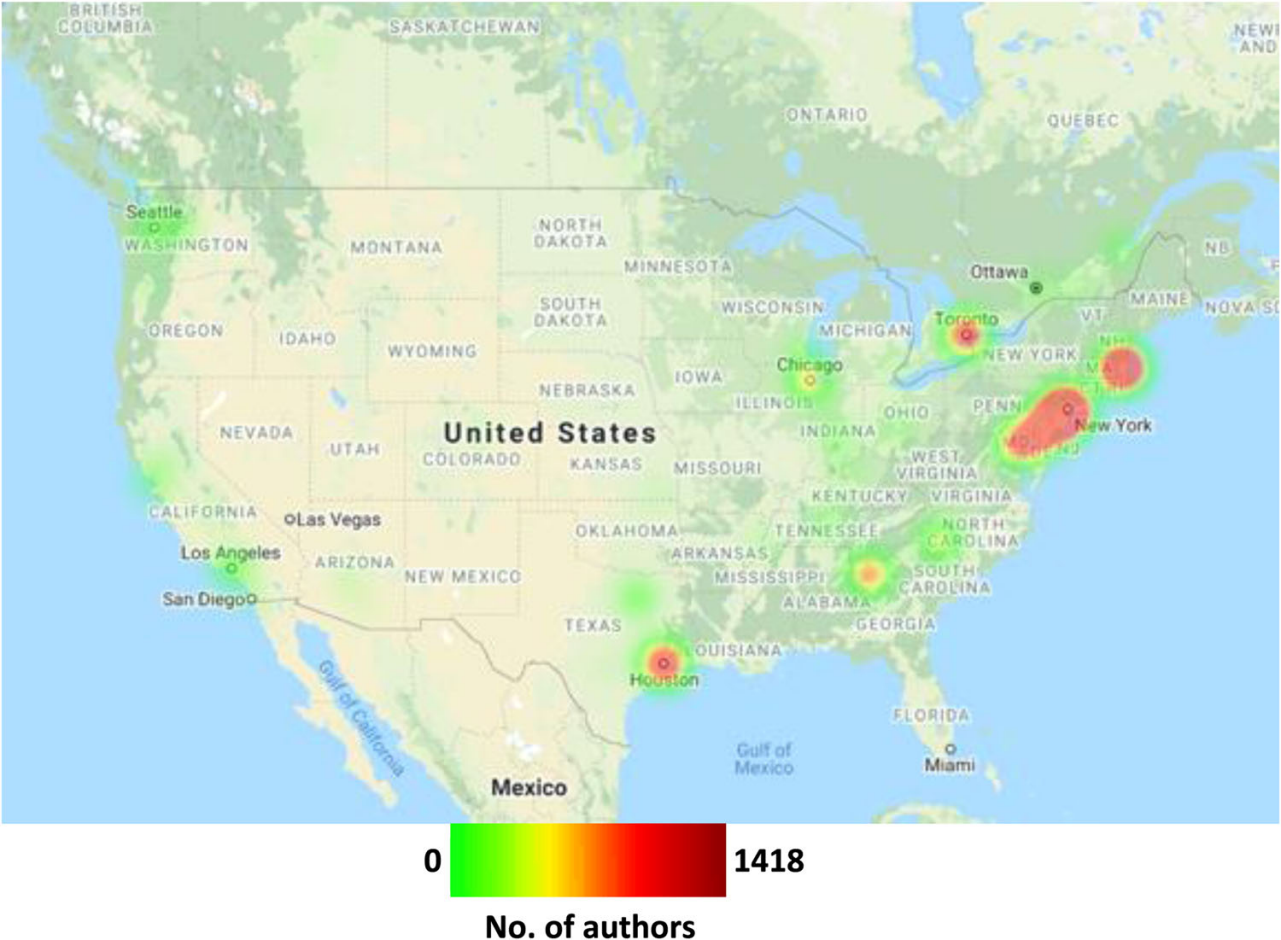
Geographical distribution of authorship by city within North America

**Figure 7:**
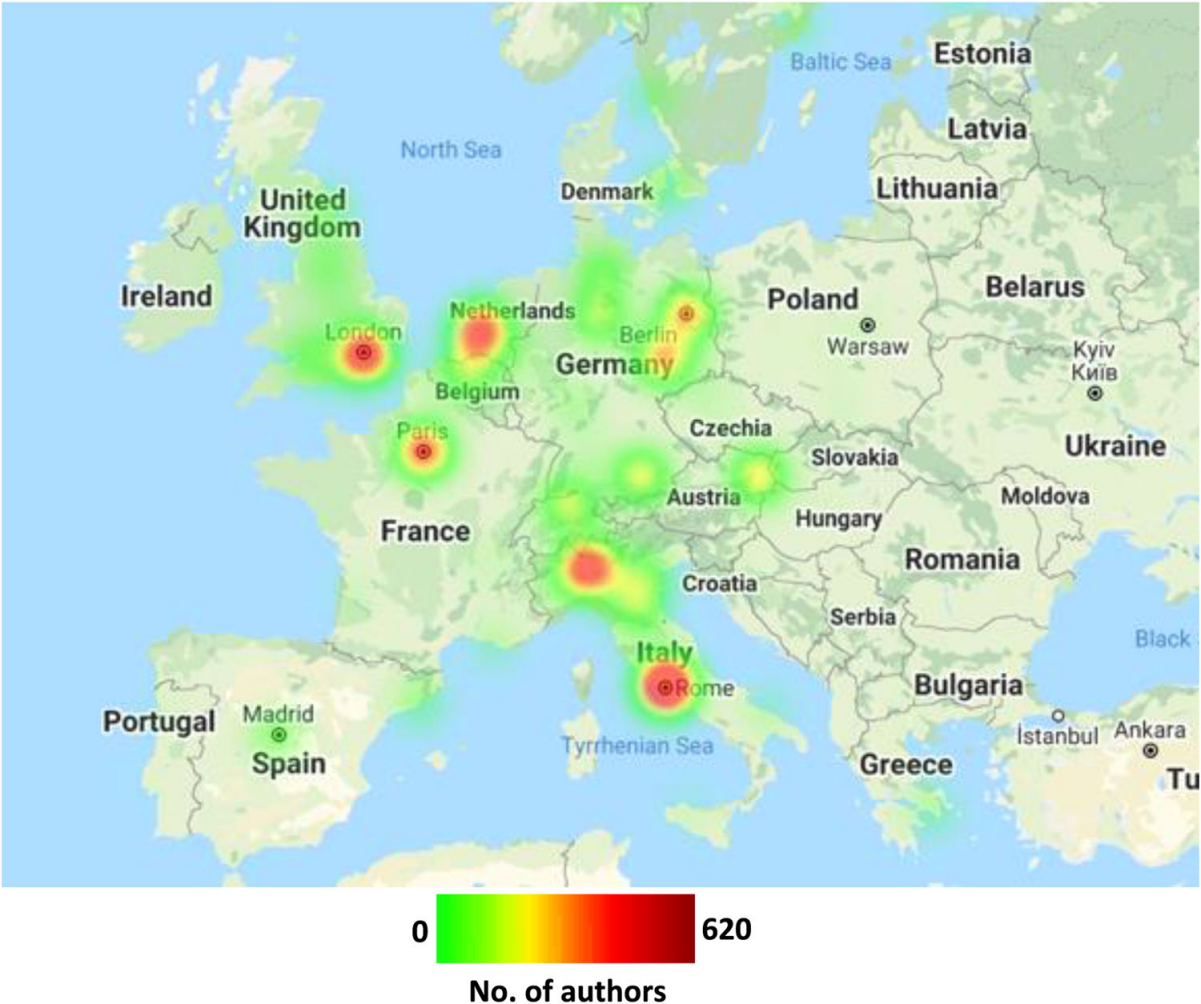
Geographical distribution of authorship by city within Europe

**Figure 8:**
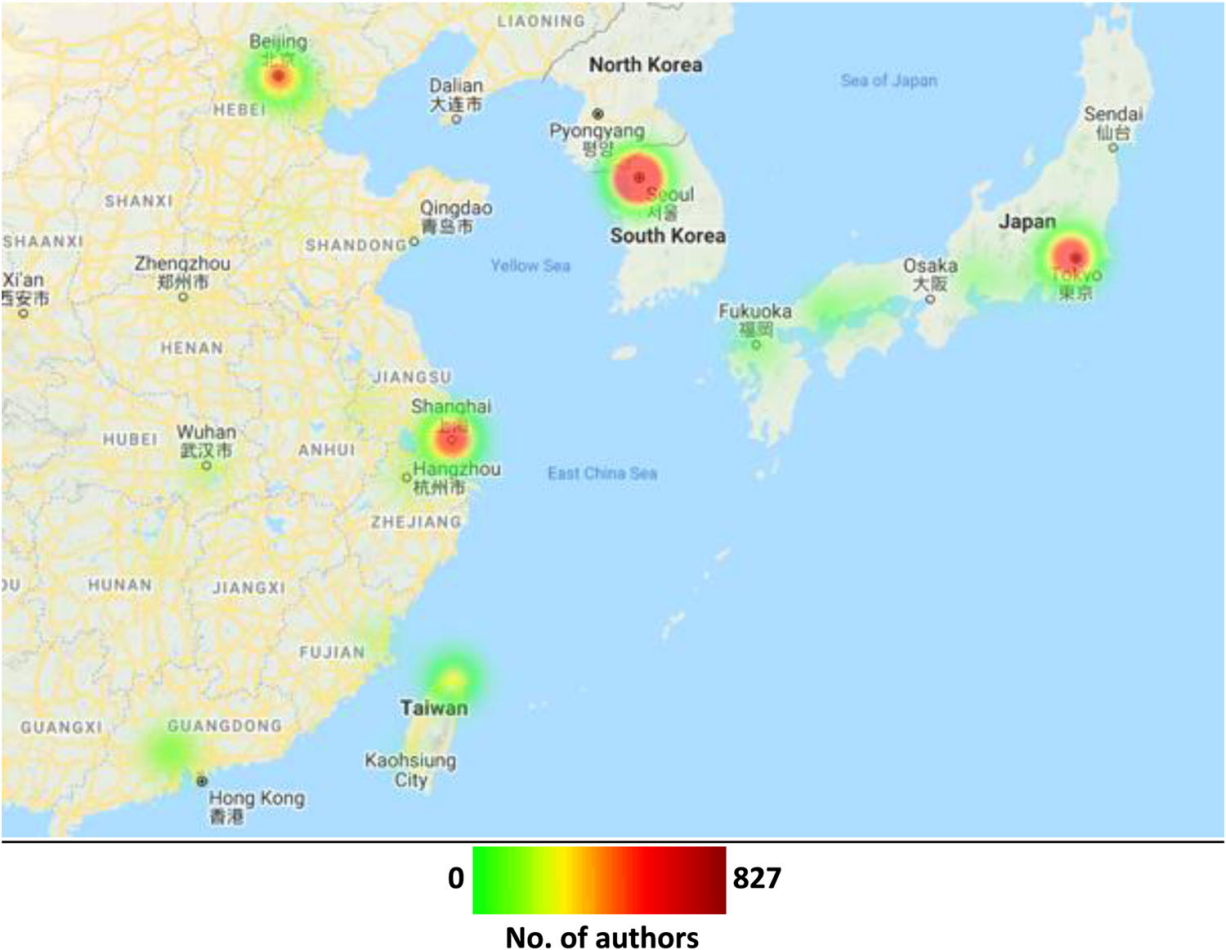
Geographical distribution of authorship by city within Asia

In 13 of the 69 countries from which authors were drawn, all authors were affiliated with a single city within that country. Cities in North American, Europe and Asia are highlighted in heat maps shown in Figures 6, 7, 8, respectively. Of the prominent North American cities, Toronto comprised 43.4% of all Canadian authorship, whilst New York City comprised 8.8% of authorship from the United States followed by Boston (7.5%) and Houston (5.5%). In Europe, London accounted for 29.2% of the United Kingdom authorship, with Rome (28.9%) and Milan (20.6%) making up large portions of Italy's authorship and Paris comprising 35.5% of France's authorship. In other areas, Seoul accounted for the vast majority of South Korea's authorship (83.6%) whilst Melbourne (44.7%) and Sydney (30.4%) accounted for large percentages of Australia's authorship. There were 1146 cities with a population greater than 500,000; 63.70% of all articles published came from authors affiliated with these cities.

In the three journals which noted highest degree (JHLT, JTCVS, ATS), the most common degree obtained by authors was MD (82.6%) followed by PhD (25.6%).

Our secondary analysis of open access journals demonstrated a lack of publications from South America and Africa. ATCS is based in Japan and featured 44.0% of authorship from Japan and 28.1% from China with only 4.1% of authors originating from the United States. JCS is European based and featured 51.4% of authorship from China, 15.3% from Europe and 8.4% from the United States. Neither journal had a single author published from South America or Africa.

## 4 DISCUSSION

To our knowledge, this is the first study to assess the geographical distribution of authorship for high impact factor cardiothoracic surgery journals. Our results show that within the leading cardiothoracic surgery journals, there is a geographical concentration of authorship, which may indicate bias towards North America and Western Europe. The most common country of origin was the United States and authors are unsurprisingly more likely to hail from large metropolitan areas. Within regions, this trend was also seen, as there were low levels of authorship from Asia outside of Japan, China, and South Korea. A minimal amount of research was published by African affiliated authors.

Papers published within the top journals in a field influence worldwide practice and shape advances in that field. When class leading journals publish more articles from certain geographical areas this introduces an element of bias within the literature. Tutarel et al.^16^ investigated the geographical distribution of contributions in the field of medical education, and also found that publications were limited to wealthy and English‐speaking countries. This is similar to the results of our analysis. Congruent findings have been published in the fields of Ophthalmology,^17^ medical informatics,^18^ and management.^19^

There are numerous potential explanations for the geographical concentration of publications in certain regions compared with others. For example, in the United States, the North‐eastern region demonstrated a significant area of focus on our heatmap which was out of proportion to the population and land size relative to the rest of the country. Though this may be contributed to by implicit bias from the journals and their editorial board members, another likely explanation is that this discrepancy in publication simply reflects the concentration of research experience, resource allocation and general infrastructure that exists in these geographical regions.^20^ In regions where the system is underfunded, intense focus must be on the delivery of clinical care with the production of clinical literature becoming a secondary priority. These issues warrant evaluation to ensure that authors from all regions have fair and equal opportunity to publish in the major journals in the field.

There is a difference in the prevalence of disease in separate countries. Interestingly, congenital heart disease prevalence is increasing in developing countries yet decreasing in wealthier countries.^21^ If major journals focus more on aspects of cardiothoracic surgery such as valve replacement surgery which is more relevant to wealthier countries, major advances in healthcare may be inequitable for patients in developing countries. Journals should assess whether their publications adequately address the major healthcare issues for all populations rather than solely higher income groups. This remains speculative though, as we did not assess the associations between country of author affiliation and specific cardiothoracic condition being studied.

The global burden of disease falls more heavily on under‐resourced populations in developing countries, with lower life expectancy, increased childhood mortality and lower levels of healthcare.^22^ Authors in developing nations are underrepresented in our sample and hence are missing opportunities for their research finding to be disseminated in the most influential journals.^23^ In the area of biomedicine, financial consideration are often taken into account to ensure journals remain profitable.^24^ If the major funding sources from these journals are located in a limited geographical location, journals may intend for their target audience to be in these areas so as to attract further funding.^25^ The geographical distribution of editorial boards also draws a parallel to the geographical distribution of publications seen in our paper and others.^20^

Conventional research funding models are subscription‐based. Individuals or institutions pay to access publications in the journal. Open access medical journals are an alternative method of publishing peer‐reviewed research, where authors pay an article processing or publication fee in order for work to be published in the journal, and contrastingly the readers are able to access publications free of charge. Open access publishing may reach a larger readership, however, smaller journals without a reputable peer‐review process may raise concerns about the quality of publications.^26^ ACTS and JCS were two open access journals we analysed for geographical distribution. Although both journals feature a greater proportion of publications from Asia, namely China and Japan, they mirror the lack of publications from South America and Africa seen in the conventional journals examined in our paper.

The journals included in our study publish both types of research and thus translatability of our findings may be limited to journals where research is limited to one stream. the distinction between basic science research and clinical research must be noted. Geography potentially affects the external validity of any clinical research performed. However, a significant proportion of the research published in major journals are basic science works.^27^ There are significant costs associated with setting up and maintaining a basic science laboratory above and beyond that of clinical science research,^28^ and the intrinsic issue is that relatively wealthy countries having more resources to dedicate to research.^29^, ^30^ This creates the scenario that funding for basic sciences research mirrors very much the geographic distribution shown in our study. This has the effect of neglecting research on problems which primarily effect lower‐income countries.^31^

There are some limitations to our work. We acknowledge that we do not know the number of papers that were submitted to these journals and without this as a denominator, it is impossible to determine the percentage acceptance from different geographical areas. This can represent an avenue of further research, so as to further elucidate contributing factors to the geographical disparity of publications in the cardiothoracic surgery literature. What our study does highlight, however, is that there is a preponderance of literature from select geographical regions. We did not analyse articles from journals with lower impact factors, although these are less likely to be widely read. We only analysed journals published in English, although we are not aware of any reputable journals in the field of cardiothoracic surgery which are published in a language other than English with an impact factor comparable to the studies included in our study. It is perhaps not surprising there is so little representation from countries that exclusively publish in journals of their own language, which could explain our findings in part. Although we have noted the differences between basic science and clinical research, our analysis did not test this difference. We did not analyse the effects of country population size on our data.

Our analysis considered did not take into account changing impact factors from 2011 to 2020. Some journals ranked in the top 5 currently may not have been ranked in the top 5 in 2011 (for example, Annals of Cardiothoracic Surgery was only first published in 2012). Similarly, we dichotomized cities with population greater than 500,000 in the year 2020 and some cities may have been above or below this threshold at different times in the past decade. Given the large volume of articles included in our paper, these discrepancies are unlikely to change the overall trends shown.

Journals already have policies in place to encourage transparency and publication of research from less well represented regions. Further possible ways to encourage this would be to inform readers of the geographical composition of editorial boards, and also the geographical distribution of publications in their journals.

## 5 CONCLUSION

The prominent cardiothoracic authorship is predominantly located in wealthy, English‐speaking countries, most commonly large metropolitan centers in the United States. This raises questions as to whether the literature adequately reflects populations in other geographical areas such as the continents of South America and Africa and rural settings. If the majority of literature is being produced in certain select geographical locations, this does raise concerns as to the generalizability of this literature on a global level, given the skill and resource disparities that exist between areas. Cardiothoracic surgery journals should consider policies which encourage publication by authors from geographical locations that are underrepresented on the world stage.
